# Frequency of Aspirin Resistance in Ischemic Stroke Patients and Healthy Controls from Colombia

**DOI:** 10.1155/2021/9924710

**Published:** 2021-05-21

**Authors:** Alejandro Roman-Gonzalez, Carlos Andrés Naranjo, Walter D. Cardona-Maya, Dionis Vallejo, Francisco Garcia, Cesar Franco, Leonor Alvarez, Luis Ignacio Tobón, Marta Ibeth López, Carolina Rua, Gabriel Bedoya, Ángela Cadavid, José Domingo Torres

**Affiliations:** ^1^Grupo de Investigación en Trombosis, Universidad de Antioquia-UdeA, Colombia; ^2^Grupo GENMOL, Universidad de Antioquia-UdeA, Colombia; ^3^Grupo Reproducción, Universidad de Antioquia-UdeA, Colombia; ^4^Departamento de Neurología, Universidad de Antioquia-UdeA, Colombia; ^5^Departamento de Neurología, Hospital Universitario San Vicente Fundación, Colombia; ^6^Instituto Neurológico de Antioquia, Colombia

## Abstract

**Objective:**

To evaluate the aspirin resistance prevalence in patients with previous ischemic cerebrovascular disease undergoing aspirin therapy for secondary prevention.

**Materials and Methods:**

Three hundred fifty patients presenting ischemic strokes and 100 healthy controls under aspirin treatment were evaluated using the optic platelet aggregation test.

**Results:**

Aspirin resistance was found in 7.4% of the patients with ischemic stroke and 4% of controls. Aspirin resistance was associated with stroke recurrence in univariate analysis (*p* = 0.004). Aspirin resistance was not associated with smoking, diabetes, or hypercholesterolemia.

**Conclusion:**

Aspirin resistance is present in Colombian patients with ischemic stroke as well as in healthy controls.

## 1. Introduction

Cerebrovascular disease is the second leading cause of death worldwide, the leading cause of death in high-income countries, and the fifth cause of death in low-income countries. Its prevalence is projected to increase in all geographical areas by 2030. Stroke is also a common cause of disability-adjusted life years and imposes a tremendous economic and social burden [1, 2].

A significant proportion of the global stroke burden is due to stroke occurrence in patients with previous strokes or transient ischemic attacks. Although the recurrence for stroke is common and the risk factors for it have been identified, there is substantial under treatment and a lack of control of these factors [3].

A mainstay for secondary prevention of stroke is tight control of risk factors and aspirin therapy [3–5]. This drug decreases the recurrence of stroke by 22% [6]. However, previous data indicate that some patients present aspirin resistance (AR) determined by the platelet function test, mainly by optic platelet aggregation [7–13]. The exact cause of this phenomenon is unclear, and genetic as well as clinical factors have been associated with AR [14]. A critical point in the AR discussion explores if AR is related to adverse outcomes compared to patients without AR. In this sense, two meta-analyses and systematic reviews including 20 studies support that AR is associated with recurrent cardiovascular events, acute coronary syndrome, and death [15, 16].

In stroke patients, the prevalence of AR is variable [17, 18]. No studies have evaluated the presence of AR in populations with a stroke from Latin American countries [19], and there is no clear evidence of the associated clinical conditions or the best treatment strategy for resistant patients.

This study is aimed at evaluating the prevalence of AR in patients with previous ischemic cerebrovascular disease undergoing aspirin therapy for secondary prevention. The patients were compared to healthy controls of similar age; AR was evaluated using optical platelet aggregation.

## 2. Materials and Methods

### 2.1. Study Population

Three hundred fifty patients with previous history of ischemic stroke were included. All patients had previously suffered one or more ischemic strokes diagnosed by a neurologist (based on clinical findings and confirmatory imaging) and were on continuous uncoated aspirin therapy for at least seven days before the sample was taken. Exclusion criteria included ingestion of nonsteroidal anti-inflammatory agents (except low dose aspirin), warfarin, ticlopidine or clopidogrel, previous surgery in the past month, paraproteinemias, thrombocytopenia, anemia, and personal or familial history suggestive of bleeding disorders. Hypertension was defined as blood pressure higher than 140/90 mmHg or treatment with any antihypertensive drug. Diabetes mellitus and hypercholesterolemia were diagnosed based on the American Diabetes Association criteria [20] and the Adult Panel Treatment III for high cholesterol levels [21], respectively. Recurrence of ischemic stroke was defined as more than one cerebrovascular event. Adherence to aspirin was evaluated by questionnaire. Optical platelet aggregation was measured. Informed consent explaining this study's objectives and ethical approval from the Research Ethics Committee of the University of Antioquia and the Hospital San Vicente Fundación were obtained.

### 2.2. Control Population

One hundred healthy individuals older than 18 years were included. None had a previous history of a cardiocerebrovascular disease or bleeding disorder. All controls received uncoated aspirin (100 mg/day) for one week, and an optical platelet aggregation test was performed before and after aspirin intake.

### 2.3. Optical Platelet Aggregation

The evaluation of platelet aggregation was performed using an optical aggregometer (AggRAM, Helena Laboratories, Texas, USA), using fresh citrated blood. Platelet-rich plasma was obtained by centrifugation of the citrated blood at 190 g for 5 min. The platelet count was adjusted to 200,000 to 300,000 platelets/mm^3^. Platelet-rich plasma (250 *μ*l) was deposited in each equipment channel, and the activators adenosine diphosphate, epinephrine, collagen, and arachidonic acid were used according to the manufacturer's instructions.

AR was defined as platelet aggregation ≥ 20% with arachidonic acid and ≥70% with adenosine diphosphate [22].

### 2.4. Statistical Analysis

The statistical analysis was performed using a proportion comparison and the Mann-Whitney Test for compared qualitative variables or quantitative variables, respectively. Differences were considered significant when *p* < 0.05. All statistical tests were performed using GraphPad Software, version 8.0 (La Jolla, CA, USA).

## 3. Results

A total of 450 individuals compromised the total population studied; 350 patients who had had a previous stroke were in the patient group, and 100 subjects were included in the control group. The patient group had a mean age of 65.6 years and was equally distributed in terms of gender; the control group was predominantly female (74%) with a mean age of 60.8 years. The control group had no history of cardiocerebrovascular events and was considered healthy given the low frequency or absence of cardiovascular risk factors. The demographic and clinical characteristics of both groups are shown in Table 1.

AR was found in 26 patients (7.4%) and 4 controls (4%) (*p* = 0.225). All of the subjects with AR defined as optic platelet aggregation > 20% with arachidonic acid and >70% with adenosine diphosphate had optic platelet aggregation > 70% with both collagen and epinephrine except for three patients. All patients with aspirin resistance had higher platelet aggregation with all the stimuli tested (*p* < 0.0001, data not shown) compared with aspirin sensitive patients.

AR was not associated with clinical risk factors of stroke. However, 46.2% of AR patients had a history of a prior stroke recurrence, with 21.3% of patients who responded to aspirin (*p* = 0.004) (Table 2).

Although no differences were found in terms of risk factors in recurrent stroke patients, a high prevalence of risk factors in this population, mainly hypertension, was found.

## 4. Discussion

The most frequently used antiplatelet therapy is aspirin, which is associated with risk factor control as the first option for secondary prophylaxis of cerebrovascular disease. However, some patients develop recurrent strokes, and AR has been postulated as an essential contributor to this therapeutic failure. Several studies support this phenomenon in patients with cardiovascular disease [23–26] and stroke [8, 10, 27–30]. The prevalence of AR is variable in these studies, but the different evaluation methods and definition criteria for AR and differences in the population studied limit the possibility of grouped analysis and reliable conclusions. The prevalence of AR in our study (7.4%) by optical platelet aggregometry is comparable to the AR published in other populations (5% to 24%) [31–33].

We found that patients with AR had more history of previous ischemic strokes than patients without AR (46.2% vs. 21.3%, *p* = 0.004). Therefore, a link between AR and stroke recurrence can be suggested. The retrospective evaluation of recurrent stroke in this study limits our conclusions of stroke recurrence and AR. However, supportive evidence from other studies also suggests that AR is associated with severe vascular events' recurrence. A prospective trial by Gum et al. [22] found that AR evaluated by optic platelet aggregation increased the risk of death, myocardial infarction, and stroke. Systematic reviews and meta-analysis confirm the higher risk of cardiovascular events in patients with AR [15, 16]. Specifically, in stroke, other researchers have found similar findings [12, 15, 16].

AR was found in healthy controls (4%). The meaning of this finding is essential given the current recommendations of the U.S Preventive Task Force for aspirin use, as primary prophylaxis in persons at risk of myocardial infarction or stroke, for the prevention of cardiovascular disease [34], raising the question of whether AR has any role in the lack of effect of aspirin for primary prevention, or if the AR test should be performed before starting aspirin therapy.

Another finding of our study was the higher frequency of AR in patients than controls, although this was not statistically significant (7.6% vs. 4% *p* = 0.225). Berrouschot et al. have suggested that some patients can develop secondary AR after continued use of this drug. Other researchers have found that AR is associated with risk factors with confirmed prothrombotic risk, such as smoking, hypercholesterolemia, and diabetes [35–37]. In the present study, an association between AR and age, gender, and cardiovascular risk factors was not observed, and these results are congruent with other studies [8, 38].

The underlying causes of aspirin resistance and therapeutic failure are not well understood, although several mechanisms may be involved, such as lack of patient adherence, underdosing, reduced absorption and increased metabolism of aspirin, biosynthesis of TXA2 from pathways not inhibited by aspirin, alternative ways involved in platelet activation not blocked by aspirin, cigarette smoking, and hypercholesterolemia.

The presence of AR in healthy controls suggests a genetic basis for this phenomenon. Several studies have examined the association of AR with a polymorphism in the COX enzyme and a number of receptors present on the surface of platelets [15, 39–41].

## 5. Conclusions

We conclude that AR is present in stroke patients from a cohort of patients in Colombia, and the frequency of detection are similar to that reported in other countries. AR is possibly associated with stroke recurrence and is a contributing factor in aspirin's therapeutic failure in stroke. However, a multifactorial scenario cannot be ruled out. The benefits of AR determination in ischemic stroke previous to and at the beginning of therapy are unknown but could be recommended given the association between AR and recurrent events in our study and the findings from other studies [15, 16]. More prospective studies with longer follow-ups are needed to determine the best treatment options for these patients.

## Figures and Tables

**Table 1:** Demographic and clinical information of patients and controls.

Variable	Patients (*n* = 350)	Controls (*n* = 100)
Age (mean and range)	65.6 (16-102)	60.8 (27-85)
Gender (male)	174 (50%)	26%
Cardiovascular risk factors		
Family history	41 (11.1%)	24 (24%)
Smoking	91 (26%)	10 (10%)
Hypertension	244 (69.7%)	12 (12%)
Hypercholesterolemia	121 (34.6%)	13 (13%)
Diabetes mellitus	62 (17.7%)	0
Atrial fibrillation	18 (5.1%)	0
Valvular disease	10 (2.9%)	0
Recurrent stroke	81 (23.1%)	0

**Table 2:** Comparison of stroke patients with and without aspirin resistance.

Variable	Aspirin sensitive (*n* = 324)	Aspirin resistant (*n* = 26)	*p* value
Age (mean and range)	65.1 (16-102)	70.0 (34-92)	0.06
Gender (male)	163 (50.3)%	12 (46.2%)	0.68
Cardiovascular risk factors			
Family history	37 (11.4%)	2 (7.7%)	0.7
Smoking	88 (27.2%)	3 (11.5%)	0.08
Hypertension	224 (69.1%)	20 (76.9%)	0.4
Hypercholesterolemia	113 (34.9%)	8 (30.8%)	0.67
Diabetes mellitus	59 (18.2%)	3 (11.5%)	0.55
Atrial fibrillation	14 (4.2%)	4 (15.4%)	0.05
Valvular disease	10 (3.1%)	0 (0%)	ND
Recurrent stroke	69 (21.3%)	12 (46.2%)	0.004

## Data Availability

The data used to support the findings of this study are available from the corresponding author upon request.
